# Introduction to the Special Issue “Emerging Trends in Combustible Tobacco and Vaping Product Use”

**DOI:** 10.3390/ijerph19094992

**Published:** 2022-04-20

**Authors:** Michael S. Dunbar, Joan S. Tucker

**Affiliations:** 1 RAND Corporation, 4750 Fifth Avenue, Suite 600, Pittsburgh, PA 15213, USA; 2 RAND Corporation, 1776 Main Street, Santa Monica, CA 90401, USA; jtucker@rand.org

Tobacco use remains a leading cause of preventable death and disease worldwide [1]. While combustible products pose the greatest and most well-established harm, the emergence of noncombustible vaping products (e.g., electronic nicotine delivery systems (ENDS) or e-cigarettes; heat-not-burn or heated tobacco products (HTPs)) has afforded greater access to a wide array of products with varying health risk profiles [2,3,4]. Such shifts in the tobacco product landscape, in concert with evolving tobacco control efforts, are driving changes in the ways in which individuals consume nicotine and tobacco via combustible, vaping, and other products. For example, the prevalence of combustible cigarette smoking has reached historic lows in the United States (U.S.) in recent years [2]. However, smoking prevalence remains high among some segments of the population compared with others [2], and the rate of vaping product use among youths and young adults [5,6] has raised serious concerns about the potential net public health impact of vaping products. Outside of the U.S., some evidence suggests that new-generation HTPs are becoming popular among former established adult cigarette smokers [7] and, potentially, adolescents [8]. Concurrent use of different types of nicotine/tobacco products (i.e., poly-tobacco use) has also become common in some population subgroups [9]. In this dynamic context, monitoring current use trends and assessing factors associated with use of combustible and noncombustible nicotine/tobacco products is critical for gauging the potential impact of different products on global public health, and for informing ongoing efforts to reduce tobacco-related harm.

This Special Issue of the International Journal of Environment Research and Public Health was developed with these factors in mind, with the goal of including a range of articles that address various facets relevant to characterizing the “emerging trends” in combustible and noncombustible nicotine/tobacco use. This Special Issue includes a diverse set of ten open access articles that focus broadly on patterns and correlates of combustible tobacco and noncombustible nicotine/tobacco product use. In this editorial letter, we discuss some of the key contributions of these studies—which employ different methods and focus on different countries, populations of interest, products, and research questions—to advance our understanding of emerging trends in product use.

Multiple studies present data on poly-tobacco use (e.g., ENDS + cigarettes; ENDS + HTPs; ENDS + cigarettes + other tobacco products) and the associated factors in adults [10,11,12]. For example, Mattingly et al. [12] leverage data from two nationally representative U.S. surveys to describe longitudinal trends in single-product and poly-product use among U.S. adults between 2014 and 2019. Findings from this study indicate increases in exclusive use of ENDS over time and decreases in poly-product use involving combustible cigarettes. Many articles in this Special Issue also point to group differences in product use patterns by tobacco use history and sociodemographic factors, such as age, sex/gender identity, sexual orientation, and race/ethnicity (e.g., [10,11,12,13,14,15,16,17]). Such findings underscore the importance of ongoing efforts to assess differences in use patterns, while product and policy contexts continue to evolve to understand and address potential health disparities across population subgroups.

Product use and/or future susceptibility among young people is of particular importance, and several studies focus specifically on youth and young adults. For example, Gaiha et al. [13] report on factors associated with future ENDS or e-cigarette product use susceptibility among a sample of U.S. adolescents and young adults. In this study, the authors identified differences in future intentions to use different types of e-cigarette devices (e.g., pod/cartridge-based products, disposable, refillable mod/tank) across sexual orientation/gender identity and racial/ethnic groups. In a longitudinal study of adolescents in the Netherlands, Hiemstra et al. [14] report on associations between personality characteristics and future use of combustible cigarettes and “alternative tobacco products” (e.g., e-cigarettes, waterpipes). In this study, factors such as hopelessness and sensation-seeking behavior were associated with future onset of both e-cigarette and combustible cigarette use, with slightly different patterns observed for other tobacco products (e.g., hookah or waterpipe). Additionally, Vogel et al. [15] report on nicotine/tobacco use history and other factors associated with perceptions and future willingness to use modern oral nicotine products in a sample of U.S. young adults. In this study, willingness to use these products was significantly higher among individuals who endorsed other nicotine/tobacco use (e.g., e-cigarettes, combustible cigarettes) and nearly half of survey respondents (49%) reported uncertainty as to whether oral nicotine pouches were less harmful than combustible cigarettes. Such findings underscore the importance of ongoing surveillance of poly-product use while the range of products continues to expand, as well as the need to communicate accurate information about relative product harms to the public.

Other studies describe patterns and correlates of nicotine/tobacco product use in specific policy environments and/or settings. For example, a study from Mistry et al. [16] reports on perceived changes in tobacco use during the COVID-19 pandemic among older adults in Rohingya refugee camps. In this article, the authors underscore the importance of surveillance activities and tobacco-control efforts to mitigate tobacco-related harm for at-risk groups during periods of crisis. In a separate study, Koyama et al. [11] present information on use of e-cigarettes, combustible cigarettes, and HTPs in a sample of individuals who report e-cigarette use in Japan, where e-cigarettes containing nicotine e-liquid have been prohibited for over a decade. The authors of this study show that, in this sample of over 4000 survey respondents, a majority (62%) reported the use of nicotine e-liquid and nearly half of the sample (49%) reported concurrent use of e-cigarettes with combustible cigarettes and/or HTPs. This illustrates the importance of—and notable challenges associated with—characterizing use patterns, product characteristics (e.g., nicotine content), and associated factors under more restrictive policy conditions.

Given the importance of marketing and promotional practices for nicotine/tobacco product uptake and continued use, two studies in this Special Issue examine these areas in more detail. A study by Miller et al. [17] used nationally representative survey data from the U.S.-based National Survey on Drug Use and Health to assess changes in cigarette brand market share between 2014 and 2019. Findings from this study suggest that menthol may have contributed to growth in market share over time for several top brands, which is particularly notable in the context of pending action on menthol cigarettes from the U.S. Food and Drug Administration. The authors also highlight a substantial increase in market share during the study’s time period for some top brands that utilized “natural” descriptor language in their advertising. In a qualitative study that leveraged data from the social media platform TikTok, Morales et al. [18] describe user-generated promotional content and the emerging online culture related to the disposable e-cigarette brand Puff Bar. Findings from this study highlight the potential role of social media platforms in driving product use trends, particularly among younger people who are disproportionately represented on some platforms, and the utility of social media data in staying on top of emerging product use trends.

Some studies also report novel data on understudied nicotine/tobacco use behaviors and device modification practices. For example, Heckman et al. [10] describe a series of pilot investigations examining cigarette relighting—that is, the practice of partially smoking, extinguishing, and subsequently relighting a cigarette. Findings from this pilot work suggest that this understudied behavior may be common among those who smoke cigarettes, particularly individuals with lower socioeconomic status, and suggest a need for more research on this phenomenon. In a qualitative study, Massey et al. [19] report on motivations for modifying ENDS devices based on focus groups with adults who use ENDS in the U.S. and highlight a number of reasons for device modifications, such as perceived satisfaction and enjoyment, controlling nicotine intake, and the use of cannabis products with ENDS. The authors emphasize the importance of shifts in device characteristics over time and point to a potential need for regulatory decision makers to consider such factors in developing and enforcing product standards to protect public health.

As nicotine/tobacco markets, products, and policies continue to change throughout the world, ongoing research is critical for informing decisions to further reduce the global burden of tobacco use. The articles included in this Special Issue highlight some of the research questions and methods that are important for monitoring and understanding emerging trends in use of different nicotine/tobacco products across population subgroups. Such diverse efforts are essential for ensuring that the state of science stays in pace with these real-world shifts—to the greatest possible extent—and that current and future research priorities and policy actions across the world are informed by the available evidence.
